# Scientific and Artful Voices of Resilience

**DOI:** 10.3389/fphar.2021.698567

**Published:** 2021-05-26

**Authors:** Pallavi R. Devchand

**Affiliations:** Department of Physiology and Pharmacology, Cumming School of Medicine, University of Calgary, Calgary, AB, Canada

**Keywords:** systems biology, inflammation, AI, resilience, rare disease

## Abstract

Resilience is a fluid trait that is triggered by personal experience. It is, arguably, a necessity for a scientist. What is it? You know it, when you see it. One thing is for certain: resilience reflects the dynamic toggle between change and an individual’s identity.

**“Nobody knows me, but I’m always there.**

**A statistic, a reminder of a world that doesn’t care”**

- UB40 *in* One in Ten, 1981

In the early 1990s, young Alan Wolffe would regularly hop on a flight from DC to the Canadian Rockies. He was test-driving his research: touting the delicate roles of nucleosome positioning in the fine-tune regulation of transcription. In those days of yore, the topic was debatable. And frankly, students in Calgary were perplexed by his energy. Here was a witty Englishman who reveled in facing a critical Calgarian crowd, knowing that the last question to him would always be the same riddle. Even when he was armed with beautiful data using the estrogen receptor activity on the vitellogenin gene of *Xenopus laevis* (Schild et al., 1993), the riddle was still asked and remained unanswered. Alan had more than data in his arsenal. He was a prolific, competitive and effective communicator – obsessed with a mission. Today, Alan’s spirit reflects in the casual acceptance of his science – I imagine he would have quipped at the business of histone regulation. How did the man fuel simple “beads-on-a-string” into a science fad that rocketed to potential of clinical relevance?

We call it customary – the passage of habits and food from one generation to another. Fascinating! to follow documented trails from the Egyptians Papyrus (Ebbell, 1937) to Guido Majno’s Healing Hand (Majno, 1975); or from a monk’s letter (Stone, 1763) to Nobel prizes (Bergstrom et al., 1982). Have you heard the English gentleman (Flower, 2013) challenge Shakespeare’s potions? Not everyone is a foodie. Louis Thomas spun biological tales of *Medusa and the Snail* (Thomas, 1974). And Gerry Weissman - oh my! – he will essay you deep into science of the humanities (Weissmann, 2006-2016). Wanna get out some? Steven J. Gould pairs well with a 22 km hike to a soft quarry in the Canadian Rockies (Gould, 1990). And if you are up for a laugh, Uncle Syd will tickle you sarcastic with his seven deadly sins (Brenner, 2019). Of course, you could simply bide time with Alan Lightman (2004). Why is it that despite Nobel prizes and landmark discoveries, these men created bandwidth for communicating their (lesser) passions?

Precious insights come from personal journeys. Paul Allen (2012) and Max Perutz (2002) remind us that even the most famous scientists are fated by human interactions. Oliver Sacks (2015) and Peter Bach (2011) went to a newspaper and won the hearts of so many suffering in silence. Mesmerizing blockbuster movies limelight our global responsibilities and gaping loopholes in our system. Constant Gardener (2005), *Ex Machina* (2014), Side Effect (2013), Eye in the Sky (2015), Little Boy and Fat Man (1989) rightfully message, “Kudos for inventing something, but - ”

You bet! There are many vested interests in the businesses of health (Devchand, 2019a). Who can ignore the bloody unicorns (Carreyrou, 2018) or the painful ones (Keefe, 2021)? There is an art to busting open a new field and influencing the culture and direction of medicine (FitzGerald, 2005; Schadt, 2005; Devchand et al., 1999). Luckily, we have a narrative of the beginning of Biotech (Hughes, 2013), an award-winning movie that institutionalized multi-scale biology as (The New Biology, 2011), and a vision for artificial intelligence to increase empathy in medicine (Topol, 2019; Devchand, 2019b). Increasingly, patient advocates seed and sustain their missions. What do you do when you are statistically disadvantaged because your disease is rare rather than common? One man’s trip with Castleman disease cracks open a cytokine storm of research with a global reach (Fajgenbaum, 2019).

In all tales, change comes with a resilient voice of awareness:

**“And I’ll tell it and think it and speak it and breathe it**

**And reflect it from the mountains**

**So all souls can see it”**

- Bob Dylan *in* The Freewheelin’, 1963

## Data Availability

The original contributions presented in the study are included in the article/Supplementary Material, further inquiries can be directed to the corresponding author.
